# Long Noncoding RNA: Shining Stars in the Immune Microenvironment of Gastric Cancer

**DOI:** 10.3389/fonc.2022.862337

**Published:** 2022-03-25

**Authors:** Xian Xiao, Wen Cheng, Guixing Zhang, Chaoran Wang, Binxu Sun, Chunyuan Zha, Fanming Kong, Yingjie Jia

**Affiliations:** ^1^ Department of Oncology, First Teaching Hospital of Tianjin University of Traditional Chinese Medicine, Tianjin, China; ^2^ National Clinical Research Center for Chinese Medicine Acupuncture and Moxibustion, Tianjin, China; ^3^ Graduate School, Tianjin University of Traditional Chinese Medicine, Tianjin, China

**Keywords:** lncRNA, immune microenvironment, gastric cancer, targeted therapeutic, tumor mircroenvironment

## Abstract

Gastric cancer (GC) is a kind of malignant tumor disease that poses a serious threat to human health. The GC immune microenvironment (TIME) is a very complex tumor microenvironment, mainly composed of infiltrating immune cells, extracellular matrix, tumor-associated fibroblasts, cytokines and chemokines, all of which play a key role in inhibiting or promoting tumor development and affecting tumor prognosis. Long non-coding RNA (lncRNA) is a non-coding RNA with a transcript length is more than 200 nucleotides. LncRNAs are expressed in various infiltrating immune cells in TIME and are involved in innate and adaptive immune regulation, which is closely related to immune escape, migration and invasion of tumor cells. LncRNA-targeted therapeutic effect prediction for GC immunotherapy provides a new approach for clinical research on the disease.

## Introduction

Gastric cancer (GC) is a kind of malignant tumor that develops from the gastric mucosa. According to the most recent International Agency for Research on Cancer (IARC) statistics, there were 1,089,000 new cases of GC and 776,000 deaths globally in 2020, making it the fourth leading cause of cancer mortality worldwide (1, 2). The pathogenesis of GC is very complex. At present, the role of Helicobacter pylori (HP) infection in the pathogenesis of GC has gradually been widely recognized. In addition, dietary influence, oncogene activation mutation and/or amplification, tumor suppressor gene mutation and/or inhibition, abnormal expression of cell cycle regulatory factors and signal molecules are all closely related to the occurrence and development of GC (3, 4). The screening and diagnostic procedures for middle and early GC include barium meal fluoroscopy, electronic gastroscopy, and serum pepsinogen (5). However, due to the hidden onset of early GC or the high cost of screening, the majority of patients with GC have been diagnosed as advanced stage (6). The main therapeutic strategies for GC include surgery, chemotherapy, and targeted therapy, however due to a lack of targets and drug resistance, these therapeutic strategies have not demonstrated promising results, particularly in patients with advanced GC. Immunotherapy for GC has received increasing attention in recent years as immune checkpoint research has developed, although various subtypes of GC patients respond differently to immunotherapy (7). Therefore, it is very important to find biomarkers for GC that are convenient for screening, diagnosis, prediction of drug efficacy and prognosis to guide the formulation of treatment strategies.

**Figure 1:**
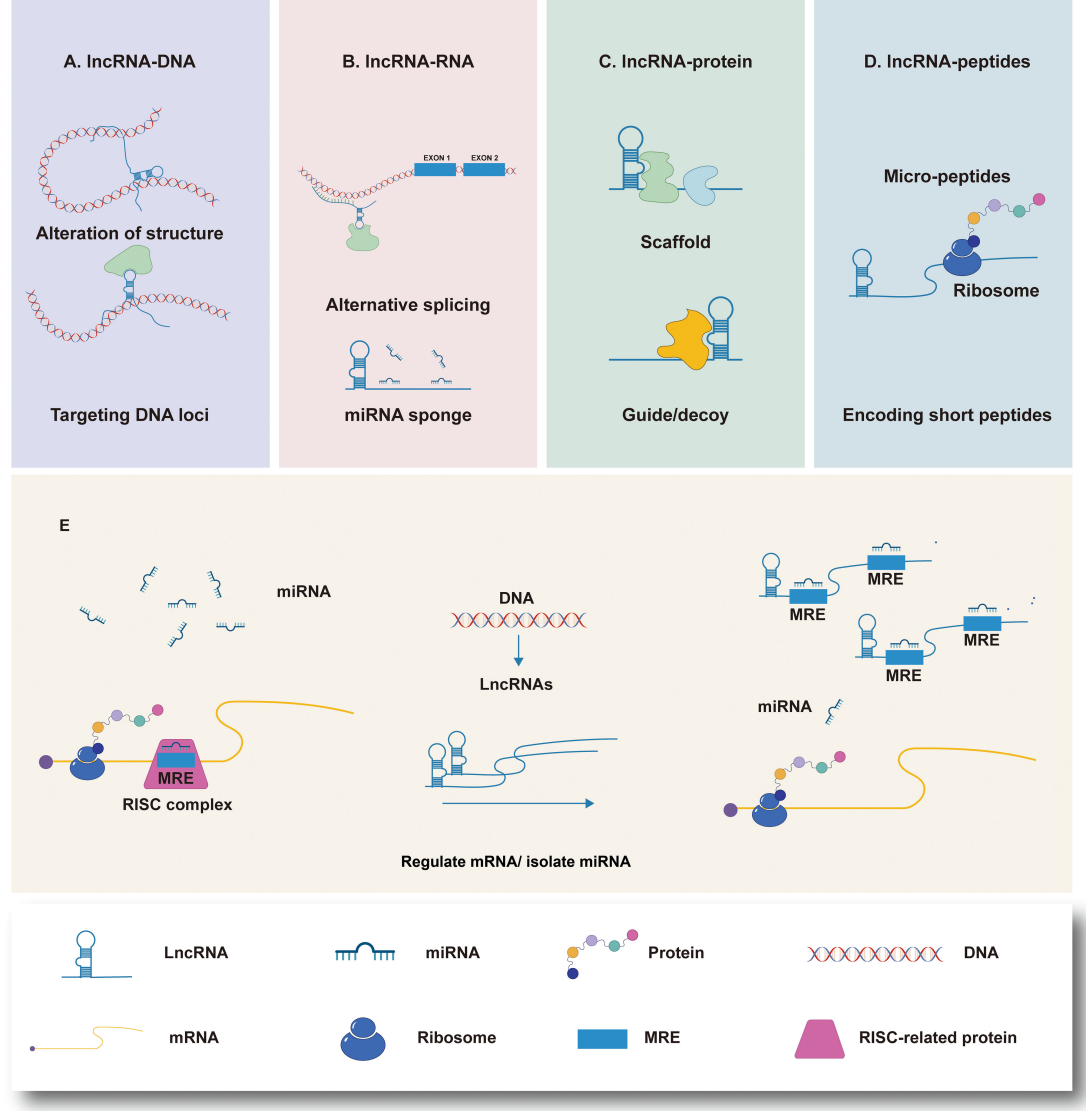
LncRNAs interactions and functions, and the mechanism of lncRNAs acting as molecular sponge. **(A)** LncRNAs regulate gene expression by affecting local chromatin structure or recruiting regulatory proteins to specific loci. **(B)** LncRNAs facilitate RNA inhibition and degradation through interacting with mRNA and miRNA to control splicing or acting as a ceRNA of miRNA. **(C)** LncRNAs can serve as molecular scaffolds, guides, or decoys for regulatory proteins to regulate protein. **(D)** A part of lncRNAs are able to encode short peptides. **(E)** MiRNAs are capable of directly binding to the matched regions of mRNAs by specific identification in a base-pairing manner, and thus inducing mRNA degradation at the post-transcriptional level by forming RNA-induced silencing complex (RISC) with related proteins such as Argonaute 2 (AGO2). LncRNAs own the miRNA response elements (MREs) which have complementary miRNA binding sites that can competitively bind to miRNAs. Therefore, lncRNAs are able to exert its biological functions by regulating the expression of mRNAs or sequestering corresponding miRNA molecules.

Long non-coding RNA (lncRNA) is a kind of non-coding RNA that has a transcript length more than 200 nucleotides (8). LncRNA is involved in a wide range of cell processes, including cell proliferation (9), differentiation (10), apoptosis (11) and immune response (12), all of which are closely related to the evolution of tumors. According to preliminary estimations from the human ENCODE project, the human genome encodes more than 28,000 distinct length lncRNA (13). Clarifying the functions of all lncRNAs is an unsolved and difficult task, although great advances have been done in recent studies on their mechanism of action. Current studies have proved that lncRNAs can regulate gene expression at the transcriptional level, post-transcriptional level and epigenetic level. Abnormal expression of lncRNAs can influence selective gene splicing, miRNA binding to mRNA, chromosome remodeling, and promoter activation through interactions with DNA, RNA, and protein, therefore impacting almost every link in gene expression (**Figure 1**) (14). Thus far, many abnormally expressed lncRNAs have been found in GC tissues. These genes can be used as oncogenes or tumor suppressor genes to regulate cell pathways, affect cell functions and participate in the generation and development of tumors (**Table 1**). Since some lncRNAs were found to be tissue-specific (34), they have been used as biomarkers for early diagnosis and prognosis of tumors by an increasing number of researchers in recent years.

**Table 1:** LncRNAs involved in GC TME.

LncRNA	Chromosomal position	Expression	Signaling pathways	Stromal cells	Clinical Significance	Reference
LINC00342	2q11.1	Upregulated in GC tissues and cell lines.	miR-545-5p/CNPY2 axis	GC	—	(15)
NCRNA00072	12q13.13	Upregulated in GC tissues and cell lines	Targeting miR-126 to active CXCR4 and RhoA	GC	—	(16)
LINC00008	11p15.5	Upregulated in GC tissues and cell lines	miR-138/E2F2 Axis	GC	—	(17)
LINC00047	11q13.1	Upregulated in GC tissues and cell lines	PI3K/AKT pathway	GC	—	(18)
LINC00256A	9q32	Upregulated in GC tissues and cell lines	FAM225A-miR-206-ADAM12 axis	GC	—	(19)
RP11-357H14.17	—	Upregulated in GC tissues and cell lines	Activating ATF2 Signaling and Enhancing Treg Cells	GC	OS	(20)
SUMO1P3	1q23.2	Upregulated in GC tissues and cell lines	Wnt/β-catenin signaling pathway	GC	—	(21)
LSINCT5	5p15.33	Upregulated in GC tissues and cell lines	Affecting the epithelial-mesenchymal transition	GC	—	(22)
LINC00001	Xq13.2	Upregulated in GC tissues and cell lines	Regulating miR-497/MACC1 axis	GC	—	(23)
LINC01540	18p11.31	Upregulated in GC tissues and cell lines	Acting as a molecular sponge of miR-378 to modulate MAPK1 expression	GC	—	(24)
HOXA-AS2	7p15.2	Upregulated in GC tissues and cell lines	Epigenetically silencing P21/PLK3/DDIT3 expression	GC	—	(25)
LINC00152	2p11.2	Upregulated in GC tissues and cell lines	EGFR-dependent pathway	GC	—	(26)
LOC554202	9p21.3	Downregulated in GC tissues	Regulate E2F1 and P15 expression	GC	—	(27)
RMRP	9p13.3	Downregulated in GC tissues	Acts as a miR-206 sponge to modulate cell cycle through regulating the expression of Cyclin D2	GC	—	(28)
GAS5	1q25.1	Downregulated in GC tissues	regulating E2F1 and P21 expression.	GC	OS, DFS	(29)
WT1-AS	11p13	Downregulated in GC tissues	Inhibit cell proliferation, migration and invasion, and increase the proportion of G0/G1 cells	GC	—	(30)
LINC00902	3q13.31	Downregulated in GC tissues	Tumor suppressors regulated by p53 and play a role by inhibiting mIR-23b	GC	DFS, DDS	(31)
LINC00023	14q32.2	Downregulated in GC tissues	p53 signaling pathway	GC	—	(32)
LINC -POU3F3	2q12.1	Upregulated in T-reg from peripheral blood of GC patients.	TGF-beta signal pathway	T-reg	—	(33)

The internal environment in which tumor cells formed and survive is known as the tumor microenvironment (TME). It plays an important role in tumor genesis and evolution. TME is mainly composed of tumor cells themselves and their surrounding fibroblasts, immune and inflammatory cells, glial cells and other stromal cells. The tumor immune microenvironment (TIME), which is composed of immune cells, is particularly important. Various immune cell components of the immune microenvironment interact closely with cancer cells during the recruitment of cytokines and tumor-related signals, and then evolve with each other to jointly promote tumor invasion and metastasis (35, 36). The components of interstitial cells involved in the regulation of TIME are complex and variable, which promote each other to form a cascade effect and jointly promote the evolution of tumor cells. The main components include tumor-associated macrophages, tumor-infiltrating lymphocytes, neutrophils, tumor-associated fibroblasts, extracellular matrix, cytokines and so on (37–40). LncRNAs molecules have an important role in tumor cell remodeling TIME and regulation of tumor cell immune escape. For example, Lnc-Tim interacts with Tim-3 to induce Bat3 release and promote CD8^+^T cell failure, resulting in hepatocellular carcinoma immune evasion (41). Lnc-sox5 promotes colorectal cancer by increasing IDO1 expression, which inhibits CD8^+^T infiltration and cytotoxicity (42). Nifk-as1 inhibits macrophage M2 polarization and endometrial cancer cell malignant phenotype by targeting miR-146a (43). The main focus of this paper was on the basic characteristics and functional roles of lncRNAs in GC TIME, as well as the immunotherapeutic potential of lncRNAs in GC treatment.

## LncRNA Is a Regulator of Immune Cells in GC TIME

### LncRNAs and GC-Associated Innate Immune Cell

GC-associated **i**nnate immune cells are mainly composed of GC-associated macrophages (CAFs), followed by dendritic cells (DCs) and natural killer cells (NK cells), etc. Through autophagocytosis, antigen recognition, cytokine synthesis and secretion, these cells play a significant role in the GC TIME.

#### LncRNAs and GC-Associated Macrophages

GC-associated macrophages infiltrated by bone marrow monocyte differentiation in TME are an important component of TIME. In TIME, macrophages are polarized into two different subtypes of macrophages by different stimuli: conventionally activated macrophages (M1 phenotype macrophages) and alternatively activated macrophages (M2 phenotype macrophages) (44, 45). M1-phenotype macrophages are activated by IFN-γ (interferon-γ), LPS (lipopolysaccharide), TNF-α (tumor necrosis factor-α), etc. After activation, immune stimulators are secreted to induce adaptive responses, as well as the secretion of reactive oxygen species and nitrogen intermediates. It is classified as anti-tumor or “good” macrophages since it is primarily involved in Th1 type immune response, monitoring tumor lesions, and resisting pathogen invasion (46). Meanwhile, M2 phenotype macrophages are usually activated in response to stimulation such as IL-4, IL-10 and IL-13. Activated M2 macrophages can release VEGF, PDGF, bFGF and other angiogenic factors as well as growth factors and matrix metalloproteinase, which can stimulate the formation of blood vessels in tumor and activate epithelial-mesenchymal transformation, invasion and metastasis of tumor cells (47). At the same time, it can also promote the formation and maintenance of tumor stem cells by increasing the expressions of IL-10 and TGF-β (transforming growth factor-β) in TIME, and reduce the expressions of IL-1, IL-6, IL-12 and TNF-α (transforming growth factor-α) (48, 49). Therefore, it is regarded as a “bad” macrophage promoting tumor.

So far, it has been proven that a variety of LncRNAs play a role in the polarization of GC-associated macrophages, hence influencing GC progression. Xie et al. found that highly expressed LncRNA ANCR in GC tissues down-regulated FoxO1 expression by promoting FoxO1 ubiquitination and degradation, and reduced IL-1β and IL-6 secretion, facilitating GC cell invasion and metastasis (50). Nie et al. found that lncRNA HCG18 up-regulated KLF4 expression by decreasing miR-875-3p in macrophages mediated by GC derived exosomes, thereby promoting polarization of M2 macrophages (51). Furthermore, a bioinformatics analysis revealed that H19, which is significantly expressed in GC, can regulate the expression of COL1A2 in sponge tissue miR-29A-3p. In GC, the H19-miR-29A-3p-COL1A2 axis can induce macrophage polarization from M1 to M2 (52). In summary, lncRNAs expressed by GC-associated macrophages or secreted by tumor cells regulate the function of GC-associated macrophages through a variety of mechanisms, further affecting the occurrence and metastasis of tumors, implying that targeting these lncRNAs in GC-associated macrophages or tumor cells may be a potential anti-tumor strategy.

#### LncRNAs and GC-Associated NK Cells

Natural killer (NK) cells, in addition to T cells, have pan-specific natural immune recognition and a rapid killing mechanism, making them an useful tool in anti-tumor therapy. Different from T cells, NK cells do not rely on the activation of antigen presenting cells to detect early signs of tumor transformation in time and respond immediately, making them the first line of host defense against tumor (53). It is worth noting that NK cells are not only killer cells, but also immunomodulatory cells. T cells and dendritic cells can be modulated by NK cells to have positive or negative impacts on tumor response in a variety of ways (54). For example, NK cells produce cytokines and chemokines, recruit dendritic cells (DCs), promote the maturation of DCs, and enhance adaptive immune response (55). Previous clinical studies have shown that NK cell killing activity and the number of intratomatous invasion are negatively correlated with GC risk and prognosis (56). This may be closely related to the effect of NK cell infiltration in maintaining tumor cell dormancy and inhibiting tumor metastasis (57).

Many LncRNAs are involved in the differentiation of NK cells, with the most well-known being the research of lnc-CD56.The expression of lnc-CD56, also known as AB128931, is significantly up-regulated in human NK cells and is closely related to the expression of typical NK cell surface marker CD56, which is involved in NK cell development (58). Tumor-infiltrating CD3+CD56+ NKT-like cells and impaired effector function in GC have been linked to immune escape and tumor progression. This may be related to the downregulation of lnc-CD56 in GC, although further research is needed to confirm this (59). In addition, Wei et al. found that lncRNA GAS5 in GC also enhanced the secretion of IFN-γ and TNF-α by regulating miR-18a, as well as the cytotoxicity of NK cells to GC, and the up-regulation of GAS5 expression may provide a new idea for anti-tumor therapy (60). Therefore, the importance of LncRNA in regulating NK cell infiltration in GC TIME cannot be ignored, and more exciting studies are expected to further confirm it.

#### LncRNAs and GC-Associated DCs Cells

DCs play an important role in antigen presentation. They are considered to be the most powerful professional antigen-presenting cells, with antigen presentation capability 100-1000 times that of macrophages and B cells (61). DCs and NK cells are both referred to be “former sentinels” of the immune response. In the immature state of DCs, they have a strong ability to devour. After phagocytic antigen, mature under the stimulation of cytokines, and then express CD80/86/40 and other costimulatory molecules, presenting the antigen to T cells to activate the downstream specific immune response (62). During tumor growth, DCs present antigen to naive T cells and memory T cells under the influence of the inflammatory environment and costimulatory signals, which leads to antigen tolerance or initiates and triggers effector T cell response (63). According to their origins and degrees of differentiation, DC cells can be classified as DC1 (myeloid DC, mDC) or DC2 (plasmacytoid DC, pDC) (64). Studies have shown that adequate density of mature DC in the tumor can prolong the survival of GC patients, and higher CD1/CD2 ratio and lower DC2 cell level are negatively correlated with the degree of tumor differentiation, degree of Foxp3^+^ Treg cells invasion and the risk of lymphatic metastasis (65, 66).

It is worth noting that studies on the regulation of lncRNAs on DCs mainly focused on HOTAIRM1 and lnc-DC genes. LncRNA HOTAIRM1 (HOX Antisense intergenicRNA myeloid 1, HOTAIRM1) was located between human HOXA1 and HOXA2 and played a functional role in regulating the expression of adjacent genes at the 3 end of HOXA cluster (67, 68). LncRNA HOTAIRM1 was found to be down-regulated during differentiation from monocytes to dendritic cells, and upregulation of HOTAIRM1 appeared to inhibit DCs maturation (69). Conversely, Lu et al. showed that the LncRNA HORAIRM1 suppressed the PI3K/AKT pathway and inhibited the development of GC by acting as a competing endogenous RNA of miR-17-5p and mediating the expression of PTEN (70). Because the outcomes of these two studies may be contradictory, more research into the specific mechanism of HOTAIRM1 in GC TIME is required. High-throughput screening analysis showed that lnc-DC was a specific regulatory gene for DC differentiation and development. Further mechanism studies showed that lnc-DC could promote DC cell maturation by activating STAT3 signaling pathway, positively regulate CD4^+^T cell differentiation to Th1 cell, and then regulate immune inflammatory response (71, 72). Unfortunately, the regulatory role of lnc-DC in immune system diseases such as Sjogren’s syndrome, multiple sclerosis, and systemic lupus erythematosus has been confirmed (73–75), but there is no report on the anti-tumor effect. We expect that future studies can further explore the role of lnc-DC in TIME. Recently, Zhu et al. found that LINC00963, which is highly expressed in GC tissues, regulates CDC5L expression and mediates DCs related anti-tumor immune response through competitive binding with miR-612, thus promoting GC progression. Therefore, targeting LINC00963 may be a promising GC treatment strategy (76).

### LncRNAs and GC-Associated Adaptive Immune Cell

Compared with innate immunity, adaptive immunity is relatively slow, but it has high specificity and memory function. Adaptive immunity consists of cellular immunity mediated by T cells and humoral immunity mediated by B cells. Nevertheless, since humoral immunity is rarely engaged in GC TIME, no studies on the role of B cells in GC TIME are currently available. Here, we principally focus on reviewing the role of T cells in GC TIME.

#### LncRNAs and GC-Associated T Cells

T cells, which are the second most common type of immune cell in tumors after macrophages, play a dual role in tumor development. Immune escape of tumor cells is usually closely related to the activation of immunosuppressive properties of T cells and the weakening of anti-tumor properties (77).

##### CD8^+^ T Cell

CD8^+^ T cells are the main T cell population in TIME and have effective anti-tumor attack effect (78). Activated CD8^+^ T differentiates into cytotoxic T lymphocytes (CTL), which have an effective anti-tumor effect by releasing perforin or promoting apoptosis, leading to direct destruction of target cells (79). In general, high levels of CD8+ T cell infiltration are linked to favorable therapeutic response and clinical outcomes in a variety of tumor tissues (80). Similarly, Lu et al. found that GC patients with a high density of CD8^+^ T cells in MSI-High GC had a higher overall survival rate than patients with low density (81). LncRNAs are currently regarded to be an important regulator of CD8+ T cell activity. LINC0152, which is up-regulated in tumor tissues and perimeters of GC patients, has been considered as an oncogene. Ou et al. found that LINC00152 inhibits the production of Th1-type chemokines CXCL9 and CXCL10 by binding to the enzymatic subunit EZH2 of PPC2, reducing the number of tumor-infiltrating CD8^+^ T cells and thereby contributing to tumor progression (82).

##### CD4^+^ T Cell

CD4+ T cells are activated primarily by MHC class II antigen recognition and serve an important regulatory role in anti-tumor immune response. It has been found that in tumor immunity, CD4^+^ T cells can activate CD8^+^ T cells through a variety of mechanisms, allowing them to differentiate into CTL while maintaining and enhancing the anti-tumor response of CTL. On the other hand, CD4+ T cells can kill tumor cells directly through the IFN- γ mechanism even in the absence of CD8+ T cells (83). Therefore, scientists regard it as a non-negligible “supporting role” in TIME.

To adapt to varied developmental and environmental conditions, naive CD4+ T cells have high plasticity and can differentiate into multi-seed cells (84). Th1, Th2 and Th17 are part of helper T (Th) cells, which are differentiated from antigen-stimulated primitive CD4+ T cells and play different anti-tumor immune functions. Th1 cells mainly secrete IFN-γ and IL-2, which activate CD8+ T cells and natural killer (NK) cells, promoting cellular immunity. To mediate humoral immunity, Th2 cells mainly secrete IL-4, IL-10, and IL-13. Th17 cells differentiate from Naïve CD4^+^ T cells induced by both TGF-β and IL-6, and they affect inflammation and progression of tumor diseases (85). LncRNA is also involved in the regulation of Th cells. According to Yao et al., high expression of lncRNAs (A2M-AS1, C2orf27A, and ZNF667-AS1) in GC tissues may act on hub ferroptosis-related genes, impair the activation of CD4^+^ T cells and Th cell infiltration, and ultimately lead to poor prognosis of GC (86). Lnc-SGK1 was shown to be significantly upregulated in GC tissue and peripheral blood, and it was linked to HP infection and a high salt diet. On another study, Yao et al. found that Lnc-SGK1 induces Th2 and Th17 differentiation while reducing Th1 differentiation through the SGK1/JunB signaling pathway, which is closely related to the poor prognosis of GC (87).

##### Treg Cells

Treg cells are a subset of CD4^+^ T cells with a significant immunosuppressive effect. At present, the most studied cells are CD4^+^CD25^+^ Treg cells, which express the transcription factor Foxp3 in their cytoplasm. Most scholars identify CD4^+^CD25^+^Foxp3^+^ T cells as Treg cells. Numerous investigations have revealed that immunosuppressive regulation of Foxp3+ Treg cells is an essential mechanism of tumor immune escape (88). Deng et al. used TGF-1 signaling to induce Foxp3+Treg cells in a hypoxic environment, which could allow dominant selection in GC to evade immune surveillance (89). Some studiesshowed that the absolute number of Foxp3^+^Treg cells in peripheral blood of patients with GC was significantly lower than that of normal controls, especially in patients with lymph node metastasis (90). Generally, LncRNA serves as an oncogene in the regulation of Treg cells. High-throughput sequencing revealed that Lnc-POU3F3 could promote the proliferation of GC cells by recruiting TGF-β protein, activating TGF-β signaling pathway and promoting the distribution of Foxp3^+^ Treg in peripheral blood T cells (33). Tang et al. found through ssGSEA analysis that LncRNA RP11-357H14.17 enhanced differentiation of Treg cells by activating the ATF2 signaling pathway, and thus played a carcinogenic role in GC (20).

**Figure 2:**
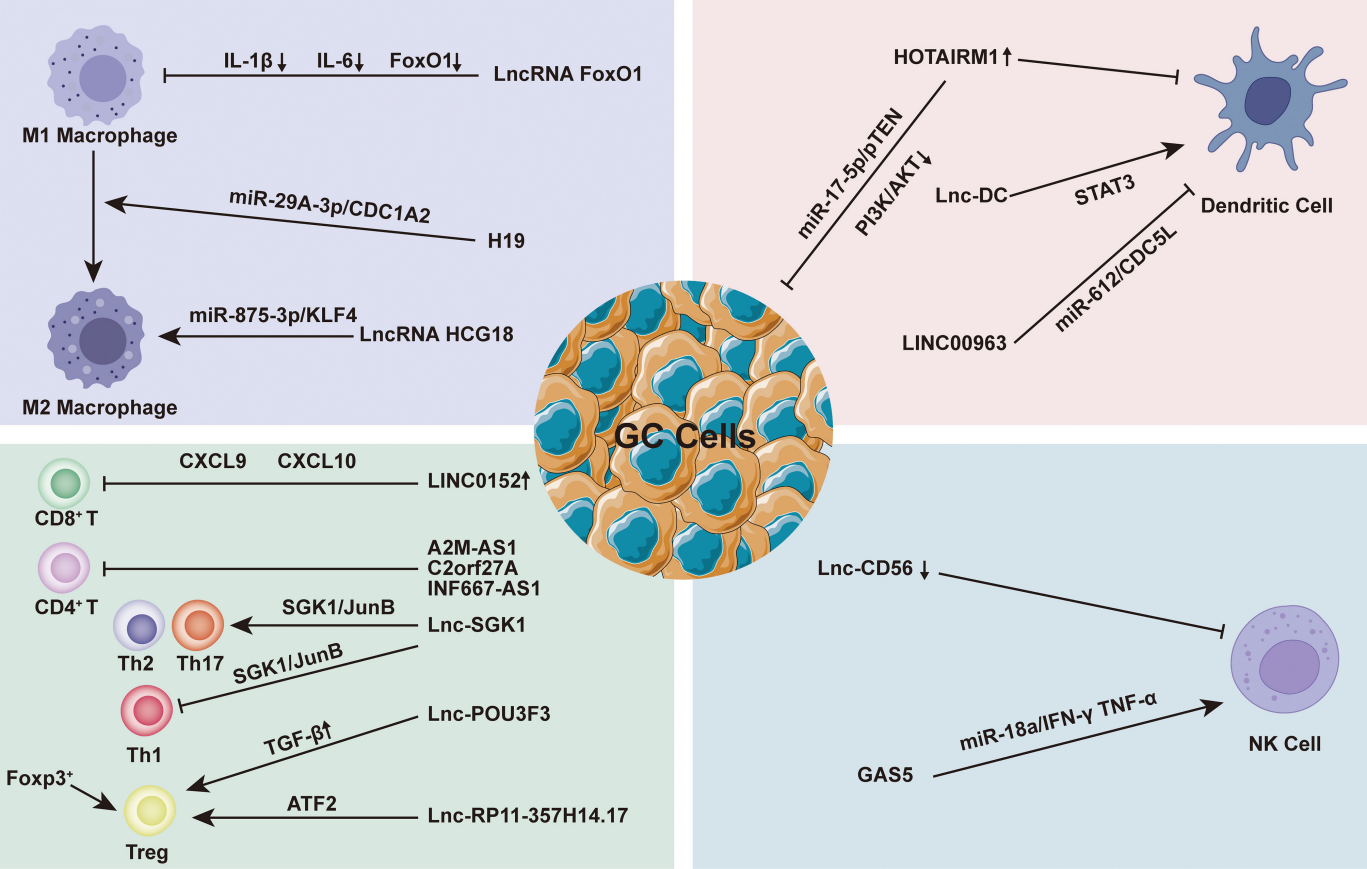
Regulation of Long non-coding RNA (lncRNAs) on immune cells in the immune microenvironment of gastric cancer (TIME). At the microenvironmental level, lncRNAs are involved in mediating and controlling various immune-cancer cell interactions. Abnormal anti-tumor immune cells [such as macrophages, dendritic cells (DC), natural killer cells (NK), and T cells and regulatory T cells (Tregs) induce the formation of immunosuppressive microenvironments, thus contributing to tumor cell metastasis.

It can be seen that lncRNAs plays a assignable role in the immune cells in TIME during the whole process of GC generation and development (**Figure 2**). However, due to the variety of lncRNA and the limited number of existing studies, further exploration is necessary.

## LncRNA Is a Regulator of Extracellular Matrix in GC TIME

Extracellular matrix (ECM) is a macromolecular substance synthesized by cells that is secreted and distributed on the cell surface or between cells. ECM is composed of basement membrane (BM) and intercellular matrix, and it serves as an important tissue barrier to prevent tumor cell metastasis. Its main components include glycosaminoglycans, proteoglycans, collagen and elastin, fibronectin (FN) and laminin (LN), the precise composition of which varies from tissue to tissue (91). ECM utilizes collagen and proteoglycans as the basic skeleton and produces a fibrous network complex on the cell surface by FN or LN directly to the cell surface membrane integrin receptor and to the cytoskeleton proteins. Through membrane integration proteins, ECM connects the inside and outside of cells, contributes in cell survival and apoptosis, affects cell shape, and regulates cell differentiation and migration. Increasing experimental and clinical observational data shows that ECM remodeling plays an important role in the precancerous cascade of GC, enhancing GC proliferation, survival, migration, invasion, and metastasis (92). For example, tenonin expression is increased in precancerous and malignant gastric epithelium, while collagen is shown to be dysregulated at more advanced stages (93, 94). ECM components and interactions are considered to have better clinical potential as prognostic biomarkers and pharmacological targets for GC.

**Figure 3:**
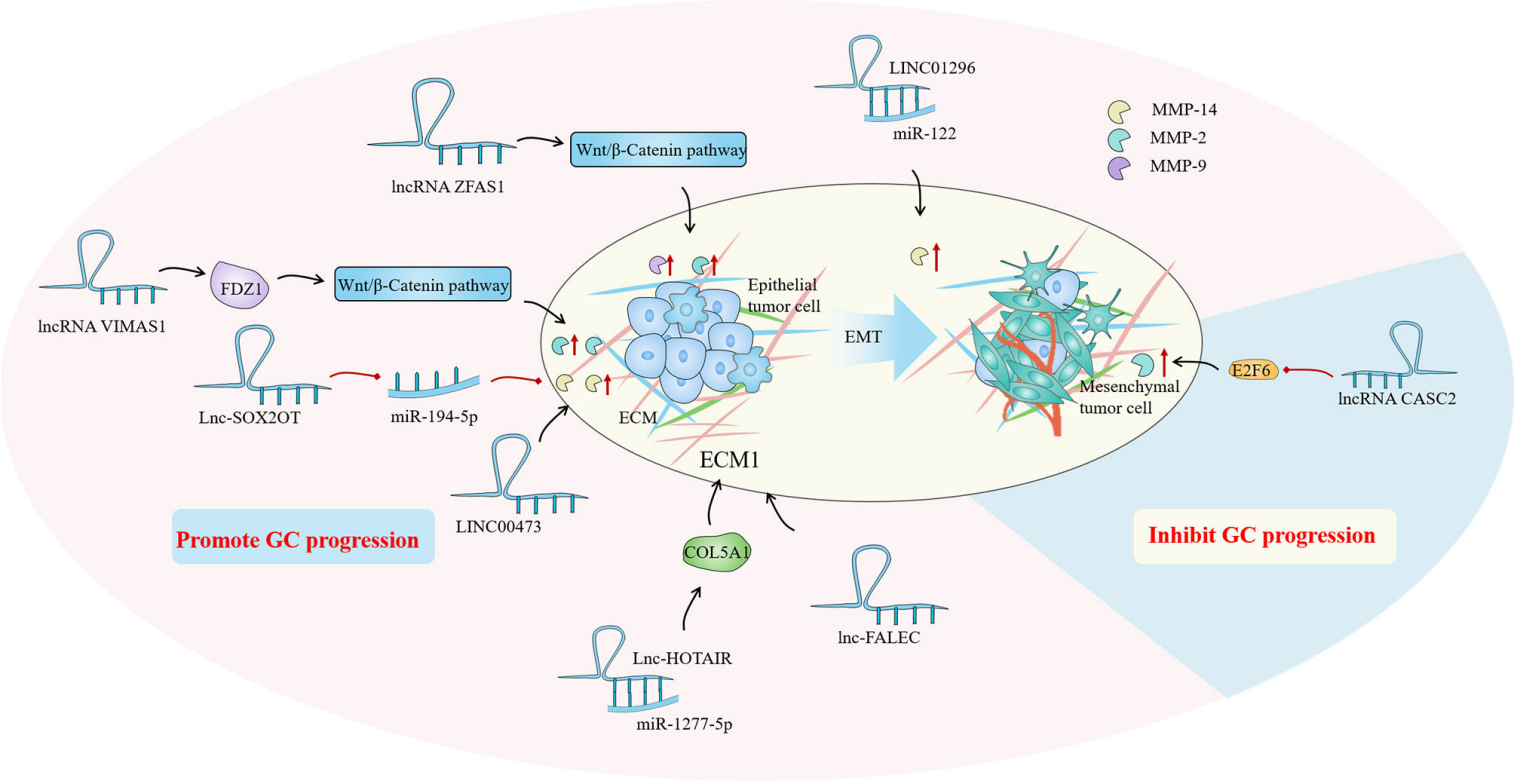
In the gastric cancer (GC) immune microenvironment (TIME), lncRNAs mainly regulate by regulating matrix metalloproteinases (MMPs) and epithelial-mesenchymal transition (EMT) to achieve the regulation of extracellular matrix (ECM), and then play a role in promoting or inhibiting GC progression.

LncRNAs play a considerable role in EMC regulation by regulating multiple targets including miRNA to achieve tissue-specific modification of ECM. Based on the evidence, we hypothesized that the modification of ECM by lncRNAs in GC is primarily focused on the regulation of matrix metalloproteinases (MMPs) and the epithelial-mesenchymal transition (EMT). MMPs are a family of Zn^2+^ and Ca^2+^ dependent endogenous proteolytic enzymes, which can be synthesized and secreted by fibroblasts, neutrophils, macrophages and tumor cells (95). The primary condition for tumor cell invasion and metastasis is degradation of ECM and destruction of BM. MMPs is the most important protease for degradation of ECM. Currently, MMPs has been found to be involved in multiple steps of tumor genesis, invasion and metastasis (95). The evolution and metastasis of GC mainly focus on MMP-2, MMP-9 and MMP-14. EMT is the biological process through which epithelial cells undergo a particular transformation into mesenchymal phenotypes. It is characterized by decreased expression of adhesion molecules (such as e-cadherin), transformation of cytoskeleton from keratin to vimentin, and mesenchymal cell morphology (96). EMT caused epithelial cells to lose their polarity, their connection to the basement membrane, and other epithelial characteristics, as well as the capacity to degrade the extracellular matrix, allowing for further migration and invasion (97). Sun et al. found that the lncRNA VIM AS1 up-regulated the expression of MMP-2 and MMP-9 proteins by regulating FDZ1 and activating the Wnt/β-catenin pathway, promoting cell proliferation, migration, invasion and epithelial-mesenchymal transformation (98). Meanwhile, LINC01296 is defined as an oncogene because it can sponge out miR-122 and then up-regulate the expression of MMP-9 protein, leading to the progression of GC (99). Moreover, Li et al. found that lncRNA CASC2 with high expression in GC tissues could reverse the regulatory effect of E2F6 gene on MMP-2, down-regulate MMP-2 expression and increase caspase-3 activity. The E2F6/CASC2 axis is expected to become a potential therapeutic target (100). Xu et al. discovered that by silencing the lncRNA ZFAS1, they could block the Wnt/-catenin signaling pathway, down-regulate the expression of MMP-2 and MMP-14 proteins, and inhibit the growth, proliferation, migration, invasion and EMT of GC cells (101). When Wei studied the SOX2OT/miR-194-5p axis in GC, they showed that the expression of miR -194-5p was negatively regulated by lnc-SOX2OT expression in GC cells. Downregulation of SOX2OT inhibited the growth of GC and the expression of MMP-2 and MMP-9 by inhibiting EMT, and it also played an effective role in anti-tumor cell metastasis (102). In addition, analysis of gene data showed that the high expression of LINC00473 in GC tissues was associated with poor histological type, advanced clinical stage, more lymph node metastasis and distant metastasis. Silencing LINC00473 can effectively regulate the expression of MMP2 and MMP9 and inhibit the migration and invasion of GC cells (103) (**Figure 3**).

Aside from the MMPs family and EMT, some ECM-related proteins have also attracted the attentions of researchers. As a collagen family protein, COL5A1 is involved in ECM formation. Bioinformatics identification showed that COL5A1 may be a key factor in many cancers, including breast cancer, ovarian cancer, lung cancer and so on (104–106). Wei et al. proved that COL5A1 may mediate the regulation of the occurrence and development of GC through its effect on ECM. Lnc-HOTAIR overexpression in GC tissues upregulated COL5A1 by sponging miR-1277-5p. ECM1 (extracellular matrix protein 1) is a glycoprotein that is involved in a variety of biological processes. A great number of studies have indicated that ECM1 can accelerate cancer development and invasion, and ECM1 overexpression has been identified as a poor prognosis indicator (107, 108). Mechanism studies have shown that ECM1 is positively correlated with the expression of lnc-FALEC in GC, and high level of ECM1 predicts shorter survival time in GC patients. Downregulation of lnc-FALEC and disruption of ECM1 expression, which significantly inhibits GC cell migration and invasion, may become potential novel therapeutic strategies (**Figure 3**).

## LncRNA Is a Regulator of Cancer Associated Fibroblasts in GC TIME

Cancer associated fibroblasts (CAFs) are the most common stromal cells in the TIME, accounting for around half of the total amount of tumor tissue cells (109). Studies in recent years have shown that CAFs mainly originate from different cells through various mechanisms, and there are three main sources of CAFs: transformation from fibroblasts (110), bone marrow mesenchymal stem cells (111), and epithelial tumor cells after EMT (112). CAFs can secrete a variety of cytokines and metabolites with tumor cells through direct contact or paracrine mode, assisting tumor cells in immune escape, promoting tumor angiogenesis, inducing tumor cells to undergo epithelial-mesenchymal transformation, promoting tumor extracellular matrix remodeling, and making the microenvironment more conducive to tumor growth (113). It has been proved that CAFs play an undeniable regulatory role in the whole process of the occurrence and evolution of GC. An analysis of the relationship between cell expression profile and clinicopathological features in TIME of 1524 patients with GC showed that the higher the number of CAFs infiltrates in TIME, the worse clinical prognosis (114). A large number of studies have shown that CAFs can directly or indirectly promote the migration and invasion of GC cells by releasing growth factors or cytokines. GC CAFs exhibit high levels of miRNA-106B, 143, and 145 expression and down-regulate miRNA-200 expression, all of which can enhance GC invasion and metastasis by various cascade pathways (115). Besides, CAFs also play a role in ECM remodeling, metabolism, and immune reprogramming. The signature function of CAFs are known for producing ECM components (such as collagen, fibronectin, proteoglycan, periostein, and tenonosin-C), which disrupt the structure of cancer tissues (116). Simultaneously, CAFs are another major source of MMPs in addition to cancer cells. All of these factors contribute to the probability of GC cell metastasis and diffusion (117).

**Figure 4:**
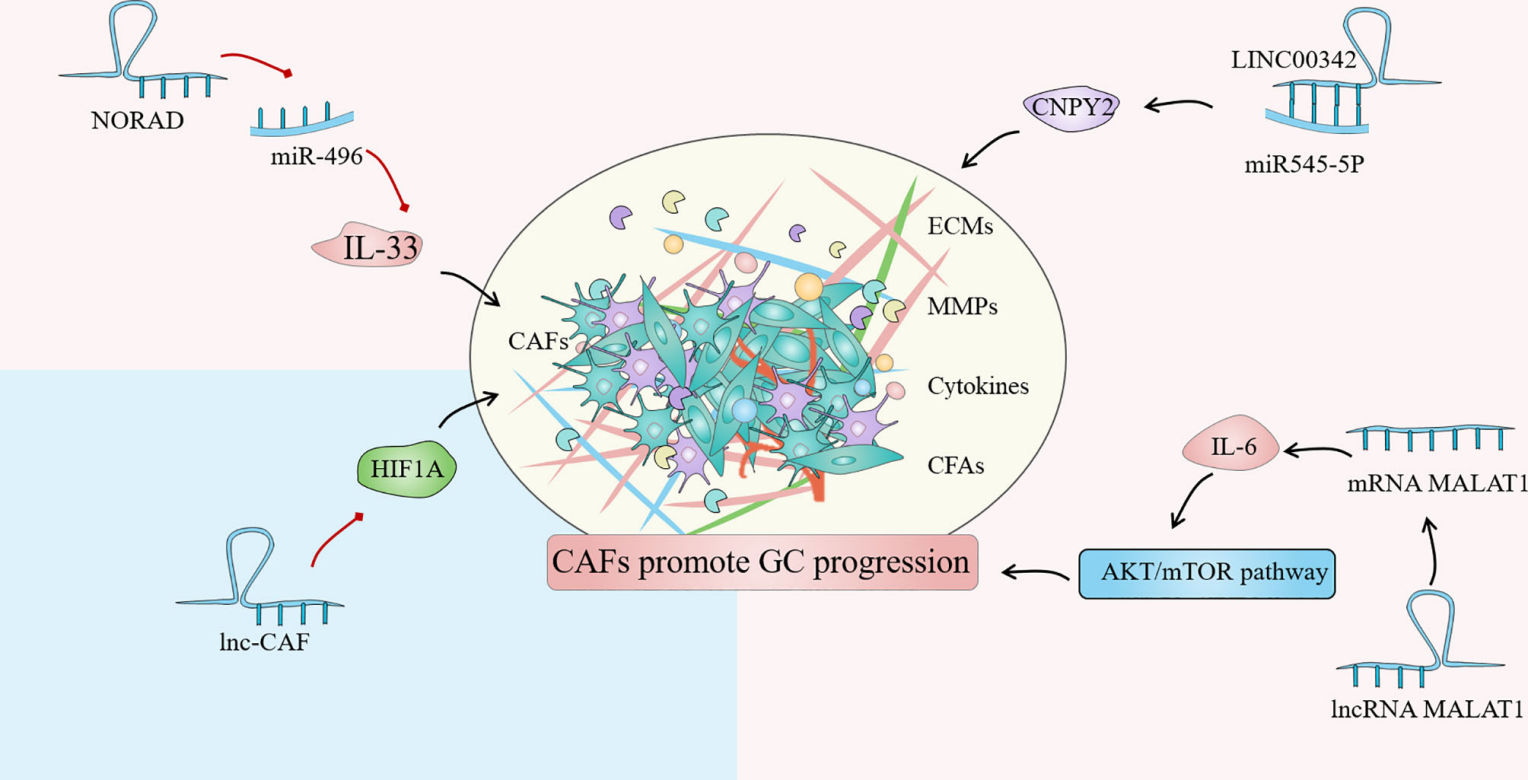
Cancer associated fibroblasts (CAFs) are one of the most common stromal cells in the gastric cancer (GC) immune microenvironment (TIME). The degree of invasion of CAFs in tissues is closely related to poor clinical prognosis. The regulation of lncRNA in GC-related CAFs is mainly manifested in the regulation of tumor cell autophagy, expression of HIF family genes, fibroblast growth factor and inflammatory factor interleukin.

Until now, the regulation of lncRNA in GC-related CAFs is mainly manifested as the regulation of autophagy of tumor cells and the expression of HIF family genes, fibroblast growth factor and inflammatory factor interleukin. Autophagy is an intracellular process that has evolved that relies on lysosomes to degrade intracellular macromolecules in bulk (118). CAFs autophagy participates in the complex metabolic and nutritional networks of tumor cells, influencing tumor progression and resistance to treatment through interactions with a variety of TIME (119). Wang et al. found that lncRNA can be used as a new regulator of autophagy, and the up-regulated lncRNA MALAT1 in GC tissues can lead to the overexpression of metastasis-associated lung adenocarcinoma transcript 1 (MALAT1), which leads to autophagy inhibition and increased IL-6 expression, thereby activating the AKT/mTOR pathway and ultimately leading to the progression of GC (120). Members of the hypoxia-inducible factor (HIF) family play a crucial part in cell hypoxia metabolism. The promotion of HIF1A and HIF2A on angiogenesis, cell metabolism, proliferation, and extracellular matrix remodeling have been demonstrated (121). By comparing the differences between GC cancer tissues and adjacent tissues, Bahramian et al. found that lnc-CAF was significantly down-regulated in cancer tissues, while the expression of HIF1A was significantly increased, which may be related to the regulation of HIF1A expression by lnc-CAF. Lnc-CAF might be one of the potential targets for cancer-targeted gene therapy (122). Additionally, Liu et al. reported that LINC00342 regulates the expression of canopy fibroblast growth factor signaling regulator 2 (CNPY2) as ceRNA by direct sponge adsorption of miR545-5P and promotes cell proliferation, colony formation, migration, and invasion *in vitro (* 15). Furthermore, noncoding RNA activated by DNA damage (NORAD) is a novel lncRNA derived from segment q11.23 of chromosome 20. Huang confirmed that NORAD could enhance the promoting effect of CAFs in GCTIME by upregulating IL-33 and targeting miR-496 (123). Overall, the regulation of lncRNAs on CAFs affects tumor progression, implying that targeting lncRNAs in CAFs and tumor cells might be a novel cancer therapy strategy (**Figure 4**).

## LncRNA Is a Regulator of Cancer Associated Cytokines in GC TIME

Cytokines are derived from immune cells and tumor cells in TIME and have diverse roles in tumor evolution and transformation *in vivo*, exerting either synergistic or antagonistic effects. They serve as a bridge for information exchange between TIME and tumor cells, despite the fact that they have no definite anti-tumor ability. The main cytokines include interleukin (IL), tumor necrosis factor (TNF), tumor growth factor (TGF), chemokine and so on (124).

The term interleukin (IL) refers to a group of soluble proteins secreted by white blood cells that can influence the functioning of other white blood cells and tissue cells. It is mainly responsible for immune cell activation and regulation, T and B cell proliferation and differentiation, and inflammatory responses *in vivo (* 125). At the moment, at least 38 IL have been identified, although there haven’t been many investigations on lncRNA-related IL. IL-21, a member of the IL-2 family, is involved in tumor biological activity and autoimmunity by binding to its receptor IL-21R (126). The IL-21/IL-21R axis has been shown to have a role in the pathogenesis and lymph node metastasis of malignant tumors by activating the JAK/STAT signaling pathway (127). Yan et al. found that IL-21R overexpression was associated with inhibition of the tumor suppressor gene miR-125a. LncRNA MALAT1 acts as a sponge for miR-125a in GC cells, and the maladjustment of the lncRNA MALAT1/miR-125a axis increaseed the risk of survival and recurrence in GC patients (128). Zhou et al. found that OLC8, a new LncRNA, was associated with IL-11 transcription. The binding of OLC8 to IL-11 greatly impaired the degradation of IL-11 mRNA. Unsurprisingly, higher IL-11 expression increased STAT3 activation and therefore contributed in the development of GC (129).

TGF mainly includes TGF-α and TGF-β, among which there are few reports on the correlation between TGF-α polymorphism and GC. The TGF-β signaling pathway plays a vital role in the genesis and development of various tumors, and this pathway has become one of the hot spots in tumor research. TGF-β1 and TGF-β2 are the core genes of this pathway, and their genetic variation has been proved to be closely related to the strength and normal down transmission of TGF-β signal, which is involved in the occurrence and development of a variety of tumors including GC (130). Zhang et al. found that the expression of LINC00665 was correlated with tumor depth, lymph node metastasis and TNM stage, and TGF-β1 was significantly reduced after LINC00665 was knocked out, which may be related to the regulation of TGF-β1 by LINC00665 (131). TGF-β1 expression is inversely linked with miR-185 expression, and the newly discovered lncRNA-XIST can reduce TGF-β1 expression by up-regulating miR-185. Therefore, the XIST/miR-185/TGF-β1 axis is also one of the primary culprits leading to the progression of GC cells (132). Likewise, several studies have found that the TGF family has a regulatory effect on LncRNA. For instance, Saito et al. discovered that TGF can activate lncRNA-ATB, promoting infiltration and metastasis in EMT through TGF-β/miR-200s/ZEB axis, leading to poor prognosis of GC (133).

**Figure 5:**
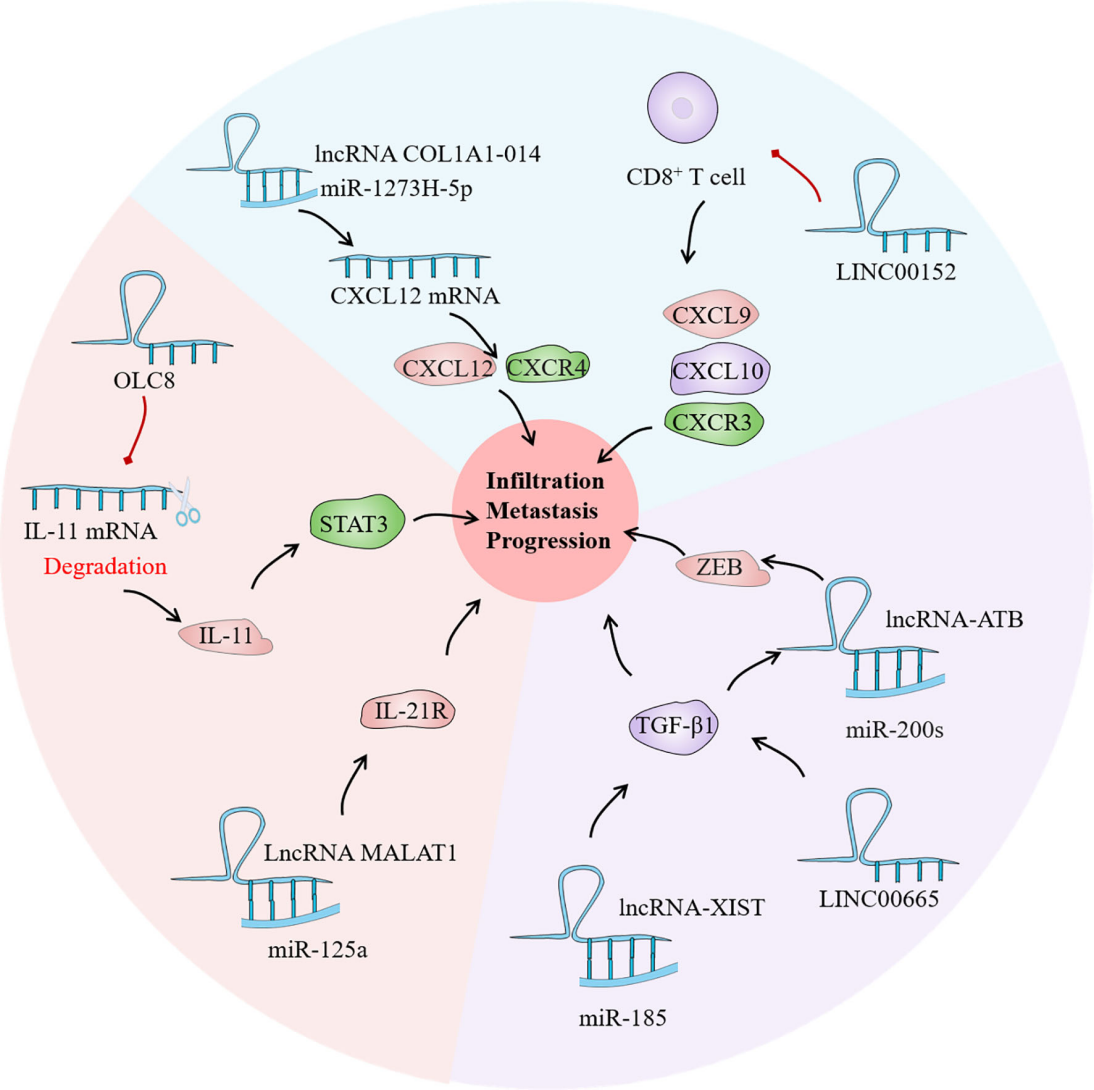
As a bridge of information exchange between gastric cancer (GC) immune microenvironment (TIME) and tumor cells, cytokines play an important role in the evolution of GC. Current studies have confirmed that lncRNA has regulatory effects on the interleukin (IL) family, tumor growth factor (TGF) and chemokines in the GC TIME, which may become a potential tumor therapeutic target.

Chemokines belong to the family of small molecule cytokine proteins, and nearly 50 chemokines have been discovered so far. All chemokine protein sequences in basic have four conservative cysteine; according to the first two cysteine differences in the relative position, it can be divided into CXC, CC, C and CX3C 4 subtypes. These chemokines are not only important in tissue differentiation and wound healing, but they are also implicated in tumor occurrence, development, invasion, and metastasis. Many investigations have currently discovered that CXC, CC, and CX3C are directly connected to GC invasion and metastasis (134). Dong et al. found that frequent up-regulation of lncRNA COL1A1-014 in GC tissues and cells increased the mRNA expression of chemokines ligand (CXCL12) in GC cells and increased the expression of CXCL12 and CXCR4 proteins through sponge absorption of miR-1273H-5p (135). Furthermore, inhibition of LINC00152 may increase the number of tumor-infiltrating CD8+ T cells and promote the expression of CXCL9, CXCL10, and C-X-C Motif chemokine receptor 3 (CXCR3) in xenograft tumors, thereby achieving the goal of tumor suppression. Collectively, LncRNAs have a significant role in tumor cytokine regulation, with complex mechanisms and various targets (**Figure 5**). Discovering effective targets of LncRNA may provide new light on targeted cancer therapy.

## Conclusion

There are interactions between cancer cells and TIME: On the one hand, cancer cells constantly secrete factors to regulate TIME, making it become a microenvironment conducive to tumor development, making TIME become a “hotbed” for cancer diffusion; on the other hand, in response to changes in environmental conditions and carcinogenic signals of tumors, TIME constantly changes during cancer development and regulates cancer progression, leading to abnormal growth, angiogenesis, metastasis and drug resistance of cancer. LncRNAs plays an important role in this process. This paper reviews the research progress of lncRNAs in GC TIME. There are several types of lncRNAs, each with a specific set of functions. LncRNAs regulate TIME cells in several ways to either inhibit or promote tumor growth and progression. LncRNAs targeting cancer immunotherapy have a wide range of potential applications. Although the application of lncRNA-based therapies has been challenging, as research advances and improves, the use of lncRNAs as therapeutic targets will contribute to the development of novel cancer treatment strategies.

## Author Contributions

XX, WC, and GZ designed the manuscript. XX wrote the manuscript. CZ, BS, and FK drew the figures and tables. YJ revised the manuscript. All authors contributed to the article and approved the submitted version.

## Funding

This study was supported by Tianjin Health Commission, Scientific research projects in key fields of traditional Chinese medicine, No. 2020008.

## Conflict of Interest

The authors declare that the research was conducted in the absence of any commercial or financial relationships that could be construed as a potential conflict of interest.

## Publisher’s Note

All claims expressed in this article are solely those of the authors and do not necessarily represent those of their affiliated organizations, or those of the publisher, the editors and the reviewers. Any product that may be evaluated in this article, or claim that may be made by its manufacturer, is not guaranteed or endorsed by the publisher.

## References

[1] Machlowska J Baj J Sitarz M Maciejewski R Sitarz R. Gastric Cancer: Epidemiology, Risk Factors, Classification, Genomic Characteristics and Treatment Strategies. Int J Mol Sci (2020) 21(11):4012. doi: 10.3390/ijms21114012 PMC7312039 32512697

[2] Bray F Ferlay J Soerjomataram I Siegel RL Torre LA Jemal A. Global Cancer Statistics 2018: GLOBOCAN Estimates of Incidence and Mortality Worldwide for 36 Cancers in 185 Countries. CA Cancer J Clin (2018) 68(6):394–424. doi: 10.3322/caac.21492 30207593

[3] Karimi P Islami F Anandasabapathy S Freedman ND Kamangar F. Gastric Cancer: Descriptive Epidemiology, Risk Factors, Screening, and Prevention. Cancer Epidemiol Biomarkers Prev (2014) 23(5):700–13. doi: 10.1158/1055-9965.EPI-13-1057 PMC4019373 24618998

[4] Nagini S. Carcinoma of the Stomach: A Review of Epidemiology, Pathogenesis, Molecular Genetics and Chemoprevention. World J Gastrointest Oncol (2012) 4(7):156–69. doi: 10.4251/wjgo.v4.i7.156 PMC3406280 22844547

[5] Lansdorp-Vogelaar I Kuipers EJ. Screening for Gastric Cancer in Western Countries. Gut (2016) 65(4):543–4. doi: 10.1136/gutjnl-2015-310356 26611232

[6] Zhao J Li X Fu L Zhang N Yang J Cai J. lncRNA LIFRAS1 Inhibits Gastric Carcinoma Cell Proliferation, Migration and Invasion by Sponging Mir4698. Mol Med Rep (2021) 23(2):153. doi: 10.3892/mmr.2020.11792 33355363 PMC7789130

[7] Vrana D Matzenauer M Neoral C Aujesky R Vrba R Melichar B. From Tumor Immunology to Immunotherapy in Gastric and Esophageal Cancer. Int J Mol Sci (2018) 20(1):21. doi: 10.3390/ijms20010013 PMC6337592 30577521

[8] Bhan A Mandal SS. LncRNA HOTAIR: A Master Regulator of Chromatin Dynamics and Cancer. Biochim Biophys Acta (2015) 1856(1):151–64. doi: 10.1016/j.bbcan.2015.07.001 PMC4544839 26208723

[9] Hung T Wang Y Lin MF Koegel AK Kotake Y Grant GD. Extensive and Coordinated Transcription of Noncoding RNAs Within Cell-Cycle Promoters. Nat Genet (2011) 43(7):621–9. doi: 10.1038/ng.848 PMC3652667 21642992

[10] Huarte M Guttman M Feldser D Garber M Koziol MJ Kenzelmann-Broz D. A Large Intergenic Noncoding RNA Induced by P53 Mediates Global Gene Repression in the P53 Response. Cell (2010) 142(3):409–19. doi: 10.1016/j.cell.2010.06.040 PMC2956184 20673990

[11] Yin Z Guan D Fan Q Su J Zheng W Ma W. lncRNA Expression Signatures in Response to Enterovirus 71 Infection. Biochem Biophys Res Commun (2013) 430(2):629–33. doi: 10.1016/j.bbrc.2012.11.101 PMC7092842 23220233

[12] Carpenter S Aiello D Atianand MK Ricci EP Gandhi P Hall LL. A Long Noncoding RNA Mediates Both Activation and Repression of Immune Response Genes. Science (2013) 341(6147):789–92. doi: 10.1126/science.1240925 PMC4376668 23907535

[13] Tragante V Moore JH Asselbergs FW. The ENCODE Project and Perspectives on Pathways. Genet Epidemiol (2014) 38(4):275–80. doi: 10.1002/gepi.21802 24723339

[14] Wang KC Chang HY. Molecular Mechanisms of Long Noncoding RNAs. Mol Cell (2011) 43(6):904–14. doi: 10.1016/j.molcel.2011.08.018 PMC3199020 21925379

[15] Liu R Yang X. LncRNA LINC00342 Promotes Gastric Cancer Progression by Targeting the miR-545-5p/CNPY2 Axis. BMC Cancer (2021) 21(1):1163. doi: 10.1186/s12885-021-08829-x 34715819 PMC8556989

[16] Xiao J Lai H Wei SH Ye ZS Gong FS Chen LC. lncRNA HOTAIR Promotes Gastric Cancer Proliferation and Metastasis *via* Targeting miR-126 to Active CXCR4 and RhoA Signaling Pathway. Cancer Med (2019) 8(15):6768–79. doi: 10.1002/cam4.1302 PMC6825996 31517442

[17] Yu J Fang C Zhang Z Zhang G Shi L Qian J. H19 Rises in Gastric Cancer and Exerts a Tumor-Promoting Function *via* miR-138/E2F2 Axis. Cancer Manag Res (2020) 12:13033–42. doi: 10.2147/CMAR.S267357 PMC7762430 33376397

[18] Zhu K Ren Q Zhao Y. lncRNA MALAT1 Overexpression Promotes Proliferation, Migration and Invasion of Gastric Cancer by Activating the PI3K/AKT Pathway. Oncol Lett (2019) 17(6):5335–42. doi: 10.3892/ol.2019.10253 PMC6507354 31186750

[19] Chen N Zhu X Zhu Y Shi J Zhang J Tang C. The Regulatory Relationship and Function of LncRNA FAM225A-miR-206-ADAM12 in Gastric Cancer. Am J Transl Res (2021) 13(8):8632–52. PMC8430187 34539984

[20] Xiaoli T Wenting W Meixiang Z Chunlei Z Chengxia H. Long Noncoding RNA RP11-357h14.17 Plays an Oncogene Role in Gastric Cancer by Activating ATF2 Signaling and Enhancing Treg Cells. BioMed Res Int (2021) 2021:6635936. doi: 10.1155/2021/6635936 34195276 PMC8181105

[21] Xu Z Ran J Gong K Hou Y Li J Guo Y. LncRNA SUMO1P3 Regulates the Invasion, Migration and Cell Cycle of Gastric Cancer Cells Through Wnt/beta-Catenin Signaling Pathway. J Recept Signal Transduct Res (2021) 41(6):574–81. doi: 10.1080/10799893.2020.1836494 33179980

[22] Qi P Lin WR Zhang M Huang D Ni SJ Zhu XL. E2F1 Induces LSINCT5 Transcriptional Activity and Promotes Gastric Cancer Progression by Affecting the Epithelial-Mesenchymal Transition. Cancer Manag Res (2018) 10:2563–71. doi: 10.2147/CMAR.S171652 PMC6089107 30127643

[23] Ma L Zhou Y Luo X Gao H Deng X Jiang Y. Long Non-Coding RNA XIST Promotes Cell Growth and Invasion Through Regulating miR-497/MACC1 Axis in Gastric Cancer. Oncotarget (2017) 8(3):4125–35. doi: 10.18632/oncotarget.13670 PMC5354817 27911852

[24] Diao L Wang S Sun Z. Long Noncoding RNA GAPLINC Promotes Gastric Cancer Cell Proliferation by Acting as a Molecular Sponge of miR-378 to Modulate MAPK1 Expression. Onco Targets Ther (2018) 11:2797–804. doi: 10.2147/OTT.S165147 PMC5957056 29785127

[25] Xie M Sun M Zhu YN Xia R Liu YW Ding J. Long Noncoding RNA HOXA-AS2 Promotes Gastric Cancer Proliferation by Epigenetically Silencing P21/PLK3/DDIT3 Expression. Oncotarget (2015) 6(32):33587–601. doi: 10.18632/oncotarget.5599 PMC4741787 26384350

[26] Zhou J Zhi X Wang L Wang W Li Z Tang J. Linc00152 Promotes Proliferation in Gastric Cancer Through the EGFR-Dependent Pathway. J Exp Clin Cancer Res (2015) 34:135. doi: 10.1186/s13046-015-0250-6 26538117 PMC4632266

[27] Nie FQ Ma S Xie M Liu YW De W Liu XH. Decreased Long Noncoding RNA MIR31HG is Correlated With Poor Prognosis and Contributes to Cell Proliferation in Gastric Cancer. Tumour Biol Jun (2016) 37(6):7693–701. doi: 10.1007/s13277-015-4644-z 26692098

[28] Shao Y Ye M Li Q Sun W Ye G Zhang X. LncRNA-RMRP Promotes Carcinogenesis by Acting as a miR-206 Sponge and is Used as a Novel Biomarker for Gastric Cancer. Oncotarget (2016) 7(25):37812–24. doi: 10.18632/oncotarget.9336 PMC5122351 27192121

[29] Sun M Jin FY Xia R Kong R Li JH Xu TP. Decreased Expression of Long Noncoding RNA GAS5 Indicates a Poor Prognosis and Promotes Cell Proliferation in Gastric Cancer. BMC Cancer (2014) 14:319. doi: 10.1186/1471-2407-14-319 24884417 PMC4022532

[30] Du T Zhang B Zhang S Jiang X Zheng P Li J. Decreased Expression of Long Non-Coding RNA WT1-AS Promotes Cell Proliferation and Invasion in Gastric Cancer. Biochim Biophys Acta (2016) 1862(1):12–9. doi: 10.1016/j.bbadis.2015.10.001 26449525

[31] Qi P Xu MD Shen XH Ni SJ Huang D Tan C. Reciprocal Repression Between TUSC7 and miR-23b in Gastric Cancer. Int J Cancer (2015) 137(6):1269–78. doi: 10.1002/ijc.29516 25765901

[32] Wei GH Wang X. lncRNA MEG3 Inhibit Proliferation and Metastasis of Gastric Cancer *via* P53 Signaling Pathway. Eur Rev Med Pharmacol Sci (2017) 21(17):3850–56. 28975980

[33] Xiong G Yang L Chen Y Fan Z. Linc-POU3F3 Promotes Cell Proliferation in Gastric Cancer *via* Increasing T-Reg Distribution. Am J Transl Res (2015) 7(11):2262–9. PMC4697706 26807174

[34] Batista PJ Chang HY. Long Noncoding RNAs: Cellular Address Codes in Development and Disease. Cell (2013) 152(6):1298–307. doi: 10.1016/j.cell.2013.02.012 PMC3651923 23498938

[35] Zhan HX Zhou B Cheng YG Xu JW Wang L Zhang GY. Crosstalk Between Stromal Cells and Cancer Cells in Pancreatic Cancer: New Insights Into Stromal Biology. Cancer Lett (2017) 392:83–93. doi: 10.1016/j.canlet.2017.01.041 28189533

[36] Gentles AJ Newman AM Liu CL Bratman SV Feng W Kim D. The Prognostic Landscape of Genes and Infiltrating Immune Cells Across Human Cancers. Nat Med Aug (2015) 21(8):938–45. doi: 10.1038/nm.3909 PMC4852857 26193342

[37] Hanahan D Weinberg RA. The Hallmarks of Cancer. Cell (2000) 100(1):57–70. doi: 10.1016/S0092-8674(00)81683-9 10647931

[38] Salgado R Denkert C Demaria S Sirtaine N Klauschen F Pruneri G. The Evaluation of Tumor-Infiltrating Lymphocytes (TILs) in Breast Cancer: Recommendations by an International TILs Working Group 2014. Ann Oncol (2015) 26(2):259–71. doi: 10.1093/annonc/mdu450 PMC6267863 25214542

[39] Ruffell B Coussens LM. Macrophages and Therapeutic Resistance in Cancer. Cancer Cell (2015) 27(4):462–72. doi: 10.1016/j.ccell.2015.02.015 PMC4400235 25858805

[40] Cirri P Chiarugi P. Cancer-Associated-Fibroblasts and Tumour Cells: A Diabolic Liaison Driving Cancer Progression. Cancer Metastasis Rev (2012) 31(1-2):195–208. doi: 10.1007/s10555-011-9340-x 22101652

[41] Ji J Yin Y Ju H Xu X Liu W Fu Q. Long Non-Coding RNA Lnc-Tim3 Exacerbates CD8 T Cell Exhaustion *via* Binding to Tim-3 and Inducing Nuclear Translocation of Bat3 in HCC. Cell Death Dis (2018) 9(5):478. doi: 10.1038/s41419-018-0528-7 29706626 PMC5924754

[42] Wu K Zhao Z Liu K Zhang J Li G Wang L. Long Noncoding RNA lnc-Sox5 Modulates CRC Tumorigenesis by Unbalancing Tumor Microenvironment. Cell Cycle (2017) 16(13):1295–301. doi: 10.1080/15384101.2017.1317416 PMC5531622 28632999

[43] Zhou YX Zhao W Mao LW Wang YL Xia LQ Cao M. Long Non-Coding RNA NIFK-AS1 Inhibits M2 Polarization of Macrophages in Endometrial Cancer Through Targeting miR-146a. Int J Biochem Cell Biol (2018) 104:25–33. doi: 10.1016/j.biocel.2018.08.017 30176290

[44] Sawa-Wejksza K Kandefer-Szerszen M. Tumor-Associated Macrophages as Target for Antitumor Therapy. Arch Immunol Ther Exp (Warsz) (2018) 66(2):97–111. doi: 10.1007/s00005-017-0480-8 28660349 PMC5851686

[45] Mantovani A Germano G Marchesi F Locatelli M Biswas SK. Cancer-Promoting Tumor-Associated Macrophages: New Vistas and Open Questions. Eur J Immunol (2011) 41(9):2522–5. doi: 10.1002/eji.201141894 21952810

[46] Singh S Mehta N Lilan J Budhthoki MB Chao F Yong L. Initiative Action of Tumor-Associated Macrophage During Tumor Metastasis. Biochim Open (2017) 4:8–18. doi: 10.1016/j.biopen.2016.11.002 29450136 PMC5801826

[47] Susen RM Bauer R Olesch C Fuhrmann DC Fink AF Dehne N. Macrophage HIF-2alpha Regulates Tumor-Suppressive Spint1 in the Tumor Microenvironment. Mol Carcinog (2019) 58(11):2127–38. doi: 10.1002/mc.23103 31436357

[48] Sica A Larghi P Mancino A Rubino L Porta C Totaro MG. Macrophage Polarization in Tumour Progression. Semin Cancer Biol (2008) 18(5):349–55. doi: 10.1016/j.semcancer.2008.03.004 18467122

[49] Kim J Bae JS. Tumor-Associated Macrophages and Neutrophils in Tumor Microenvironment. Mediators Inflamm (2016) 2016:6058147. doi: 10.1155/2016/6058147 26966341 PMC4757693

[50] Xie C Guo Y Lou S. LncRNA ANCR Promotes Invasion and Migration of Gastric Cancer by Regulating FoxO1 Expression to Inhibit Macrophage M1 Polarization. Dig Dis Sci (2020) 65(10):2863–72. doi: 10.1007/s10620-019-06019-1 31894487

[51] Xin L Wu Y Liu C Zeng F Wang JL Wu DZ. Exosome-Mediated Transfer of lncRNA HCG18 Promotes M2 Macrophage Polarization in Gastric Cancer. Mol Immunol (2021) 140:196–205. doi: 10.1016/j.molimm.2021.10.011 34735868

[52] Nie K Zheng Z Wen Y Pan J Liu Y Jiang X. A Novel ceRNA Axis Involves in Regulating Immune Infiltrates and Macrophage Polarization in Gastric Cancer. Int Immunopharmacol (2020) 87:106845. doi: 10.1016/j.intimp.2020.106845 32763781

[53] Gonzalez H Hagerling C Werb Z. Roles of the Immune System in Cancer: From Tumor Initiation to Metastatic Progression. Genes Dev (2018) 32(19-20):1267–84. doi: 10.1101/gad.314617.118 PMC6169832 30275043

[54] Hanson HL Donermeyer DL Ikeda H White JM Shankaran V Old LJ. Eradication of Established Tumors by CD8+ T Cell Adoptive Immunotherapy. Immunity (2000) 13(2):265–76. doi: 10.1016/S1074-7613(00)00026-1 10981969

[55] Matsushita H Vesely MD Koboldt DC Rickert CG Uppaluri R Magrini VJ. Cancer Exome Analysis Reveals a T-Cell-Dependent Mechanism of Cancer Immunoediting. Nature (2012) 482(7385):400–4. doi: 10.1038/nature10755 PMC3874809 22318521

[56] Peske JD Woods AB Engelhard VH. Control of CD8 T-Cell Infiltration Into Tumors by Vasculature and Microenvironment. Adv Cancer Res (2015) 128:263–307. doi: 10.1016/bs.acr.2015.05.001 26216636 PMC4638417

[57] Lu X Yang L Yao D Wu X Li J Liu X. Tumor Antigen-Specific CD8(+) T Cells Are Negatively Regulated by PD-1 and Tim-3 in Human Gastric Cancer. Cell Immunol (2017) 313:43–51. doi: 10.1016/j.cellimm.2017.01.001 28110884

[58] Ou J Lei P Yang Z Yang M Luo L Mo H. LINC00152 Mediates CD8(+) T-Cell Infiltration in Gastric Cancer Through Binding to EZH2 and Regulating the CXCL9, 10/CXCR3 Axis. J Mol Histol (2021) 52(3):611–20. doi: 10.1007/s10735-021-09967-z 33709190

[59] Binnewies M Mujal AM Pollack JL Combes AJ Hardison EA Barry KC. Unleashing Type-2 Dendritic Cells to Drive Protective Antitumor CD4(+) T Cell Immunity. Cell (2019) 177(3):556–71.e16. doi: 10.1016/j.cell.2019.02.005 30955881 PMC6954108

[60] Hilligan KL Ronchese F. Antigen Presentation by Dendritic Cells and Their Instruction of CD4+ T Helper Cell Responses. Cell Mol Immunol (2020) 17(6):587–99. doi: 10.1038/s41423-020-0465-0 PMC7264306 32433540

[61] Tosolini M Kirilovsky A Mlecnik B Fredriksen T Mauger S Bindea G. Clinical Impact of Different Classes of Infiltrating T Cytotoxic and Helper Cells (Th1, Th2, Treg, Th17) in Patients With Colorectal Cancer. Cancer Res (2011) 71(4):1263–71. doi: 10.1158/0008-5472.CAN-10-2907 21303976

[62] Yao F Zhan Y Pu Z Lu Y Chen J Deng J. LncRNAs Target Ferroptosis-Related Genes and Impair Activation of CD4(+) T Cell in Gastric Cancer. Front Cell Dev Biol (2021) 9:797339. doi: 10.3389/fcell.2021.797339 34966745 PMC8710671

[63] Yao Y Jiang Q Jiang L Wu J Zhang Q Wang J. Lnc-SGK1 Induced by Helicobacter Pylori Infection and Highsalt Diet Promote Th2 and Th17 Differentiation in Human Gastric Cancer by SGK1/Jun B Signaling. Oncotarget (2016) 7(15):20549–60. doi: 10.18632/oncotarget.7823 PMC4991474 26942879

[64] Wang Y Ma Y Fang Y. Regulatory T Cell: A Protection for Tumour Cells. J Cell Mol Med (2012) 16(3):425–36. doi: 10.1111/j.1582-4934.2011.01437.x PMC3822920 21895966

[65] Deng B Zhu JM Wang Y Liu TT Ding YB Xiao WM. Intratumor Hypoxia Promotes Immune Tolerance by Inducing Regulatory T Cells *via* TGF-Beta1 in Gastric Cancer. PloS One (2013) 8(5):e63777. doi: 10.1371/journal.pone.0063777 23723999 PMC3664556

[66] Feichtenbeiner A Haas M Buttner M Grabenbauer GG Fietkau R Distel LV. Critical Role of Spatial Interaction Between CD8(+) and Foxp3(+) Cells in Human Gastric Cancer: The Distance Matters. Cancer Immunol Immunother (2014) 63(2):111–9. doi: 10.1007/s00262-013-1491-x PMC11029441 24170095

[67] Demaria O Cornen S Daeron M Morel Y Medzhitov R Vivier E. Harnessing Innate Immunity in Cancer Therapy. Nature (2019) 574(7776):45–56. doi: 10.1038/s41586-019-1593-5 31578484

[68] Malmberg KJ Carlsten M Bjorklund A Sohlberg E Bryceson YT Ljunggren HG. Natural Killer Cell-Mediated Immunosurveillance of Human Cancer. Semin Immunol (2017) 31:20–9. doi: 10.1016/j.smim.2017.08.002 28888619

[69] Mahmood S Upreti D Sow I Amari A Nandagopal S Kung SK. Bidirectional Interactions of NK Cells and Dendritic Cells in Immunotherapy: Current and Future Perspective. Immunother (2015) 7(3):301–8. doi: 10.2217/imt.14.122 25804481

[70] Li B Jiang Y Li G Fisher GA Jr. Li R. Natural Killer Cell and Stroma Abundance Are Independently Prognostic and Predict Gastric Cancer Chemotherapy Benefit. JCI Insight (2020) 5(9):e136570. doi: 10.1172/jci.insight.136570 PMC7253031 32229725

[71] Correia AL Guimaraes JC Auf der Maur P De Silva D Trefny MP Okamoto R. Hepatic Stellate Cells Suppress NK Cell-Sustained Breast Cancer Dormancy. Nature (2021) 594(7864):566–71. doi: 10.1038/s41586-021-03614-z 34079127

[72] Mace EM Gunesch JT Dixon A Orange JS. Human NK Cell Development Requires CD56-Mediated Motility and Formation of the Developmental Synapse. Nat Commun (2016) 7:12171. doi: 10.1038/ncomms12171 27435370 PMC4961740

[73] Peng LS Mao FY Zhao YL Wang TT Chen N Zhang JY. Altered Phenotypic and Functional Characteristics of CD3+CD56+ NKT-Like Cells in Human Gastric Cancer. Oncotarget (2016) 7(34):55222–30. doi: 10.18632/oncotarget.10484 PMC5342413 27409423

[74] Wei MF Gu ZS Zheng LL Zhao MX Wang XJ. Long Non-Coding RNA GAS5 Promotes Natural Killer Cell Cytotoxicity Against Gastric Cancer by Regulating miR-18a. Neoplasma (2020) 67(5):1085–93. doi: 10.4149/neo_2020_191014N1034 32538667

[75] Levin D Constant S Pasqualini T Flavell R Bottomly K. Role of Dendritic Cells in the Priming of CD4+ T Lymphocytes to Peptide Antigen In Vivo. J Immunol (1993) 151(12):6742–50. 7903097

[76] Hashemi V Farhadi S Ghasemi Chaleshtari M Seashore-Ludlow B Masjedi A Hojjat-Farsangi M. Nanomedicine for Improvement of Dendritic Cell-Based Cancer Immunotherapy. Int Immunopharmacol (2020) 83:106446. doi: 10.1016/j.intimp.2020.106446 32244048

[77] Gardner A Ruffell B. Dendritic Cells and Cancer Immunity. Trends Immunol (2016) 37(12):855–65. doi: 10.1016/j.it.2016.09.006 PMC5135568 27793569

[78] Rissoan MC Soumelis V Kadowaki N Grouard G Briere F de Waal Malefyt R. Reciprocal Control of T Helper Cell and Dendritic Cell Differentiation. Science (1999) 283(5405):1183–6. doi: 10.1126/science.283.5405.1183 10024247

[79] Ananiev J Gulubova MV Manolova IM. Prognostic Significance of CD83 Positive Tumor-Infiltrating Dendritic Cells and Expression of TGF-Beta 1 in Human Gastric Cancer. Hepatogastroenterology (2011) 58(110-111):1834–40. doi: 10.5754/hge10320 22086706

[80] Li F Sun Y Huang J Xu W Liu J Yuan Z. CD4/CD8 + T Cells, DC Subsets, Foxp3, and IDO Expression Are Predictive Indictors of Gastric Cancer Prognosis. Cancer Med (2019) 8(17):7330–44. doi: 10.1002/cam4.2596 PMC6885892 31631566

[81] Prensner JR Chinnaiyan AM. The Emergence of lncRNAs in Cancer Biology. Cancer Discov (2011) 1(5):391–407. doi: 10.1158/2159-8290.CD-11-0209 22096659 PMC3215093

[82] Zhang X Lian Z Padden C Gerstein MB Rozowsky J Snyder M. A Myelopoiesis-Associated Regulatory Intergenic Noncoding RNA Transcript Within the Human HOXA Cluster. Blood (2009) 113(11):2526–34. doi: 10.1182/blood-2008-06-162164 PMC2656274 19144990

[83] Xin J Li J Feng Y Wang L Zhang Y Yang R. Downregulation of Long Noncoding RNA HOTAIRM1 Promotes Monocyte/Dendritic Cell Differentiation Through Competitively Binding to Endogenous miR-3960. Onco Targets Ther (2017) 10:1307–15. doi: 10.2147/OTT.S124201 PMC5338958 28280365

[84] Lu R Zhao G Yang Y Jiang Z Cai J Zhang Z. Long Noncoding RNA HOTAIRM1 Inhibits Cell Progression by Regulating miR-17-5p/ PTEN Axis in Gastric Cancer. J Cell Biochem (2019) 120(4):4952–65. doi: 10.1002/jcb.27770 30302796

[85] Zhou L Zhu Y Sun D Zhang Q. Emerging Roles of Long Non-Coding RNAs in The Tumor Microenvironment. Int J Biol Sci (2020) 16(12):2094–103. doi: 10.7150/ijbs.44420 PMC7294937 32549757

[86] Zhang W Zhou Y Ding Y. Lnc-DC Mediates the Over-Maturation of Decidual Dendritic Cells and Induces the Increase in Th1 Cells in Preeclampsia. Am J Reprod Immunol (2017) 77(6):e12647. doi: 10.1111/aji.12647 28185352

[87] Chen Y Chen Y Zu B Liu J Sun L Ding C. Identification of Long Noncoding RNAs lnc-DC in Plasma as a New Biomarker for Primary Sjogren's Syndrome. J Immunol Res (2020) 2020:9236234. doi: 10.1155/2020/9236234 33123604 PMC7585659

[88] Shaker OG Mahmoud RH Abdelaleem OO Ibrahem EG Mohamed AA Zaki OM. LncRNAs, MALAT1 and lnc-DC as Potential Biomarkers for Multiple Sclerosis Diagnosis. Biosci Rep (2019) 39(1):BSR20181335. doi: 10.1042/BSR20181335 30514825 PMC6331681

[89] Wu GC Li J Leng RX Li XP Li XM Wang DG. Identification of Long Non-Coding RNAs GAS5, Linc0597 and lnc-DC in Plasma as Novel Biomarkers for Systemic Lupus Erythematosus. Oncotarget (2017) 8(14):23650–63. doi: 10.18632/oncotarget.15569 PMC5410334 28423570

[90] Zhu H Tang JH Zhang SM Qian JP Ling X Wu XY. Long Noncoding RNA LINC00963 Promotes CDC5L-Mediated Malignant Progression in Gastric Cancer. Onco Targets Ther (2020) 13:12999–3013. doi: 10.2147/OTT.S274708 PMC7764734 33376349

[91] Mouw JK Ou G Weaver VM. Extracellular Matrix Assembly: A Multiscale Deconstruction. Nat Rev Mol Cell Biol (2014) 15(12):771–85. doi: 10.1038/nrm3902 PMC4682873 25370693

[92] Moreira AM Pereira J Melo S Fernandes MS Carneiro P Seruca R. The Extracellular Matrix: An Accomplice in Gastric Cancer Development and Progression. Cells (2020) 9(2):394. doi: 10.3390/cells9020394 PMC7072625 32046329

[93] Tiitta O Sipponen P Gould V Virtanen I. Tenascin Expression in Inflammatory, Dysplastic and Neoplastic Lesions of the Human Stomach. Virchows Arch (1994) 425(4):369–74. doi: 10.1007/BF00189574 7529617

[94] Zhang QN Zhu HL Xia MT Liao J Huang XT Xiao JW. A Panel of Collagen Genes Are Associated With Prognosis of Patients With Gastric Cancer and Regulated by microRNA-29c-3p: An Integrated Bioinformatics Analysis and Experimental Validation. Cancer Manag Res (2019) 11:4757–72. doi: 10.2147/CMAR.S198331 PMC6538884 31213898

[95] Fink K Boratynski J. The Role of Metalloproteinases in Modification of Extracellular Matrix in Invasive Tumor Growth, Metastasis and Angiogenesis. Postepy Hig Med Dosw (Online) (2012) 66:609–28. doi: 10.5604/17322693.1009705 23001203

[96] Baj J Korona-Glowniak I Forma A Maani A Sitarz E Rahnama-Hezavah M. Mechanisms of the Epithelial-Mesenchymal Transition and Tumor Microenvironment in Helicobacter Pylori-Induced Gastric Cancer. Cells (2020) 9(4):1055. doi: 10.3390/cells9041055 PMC7225971 32340207

[97] Bakir B Chiarella AM Pitarresi JR Rustgi AK. EMT, MET, Plasticity, and Tumor Metastasis. Trends Cell Biol (2020) 30(10):764–76. doi: 10.1016/j.tcb.2020.07.003 PMC7647095 32800658

[98] Sun JG Li XB Yin RH Li XF. lncRNA VIMAS1 Promotes Cell Proliferation, Metastasis and Epithelialmesenchymal Transition by Activating the Wnt/betacatenin Pathway in Gastric Cancer. Mol Med Rep (2020) 22(6):4567–78. doi: 10.3892/mmr.2020.11577 PMC7646824 33173977

[99] Qin QH Yin ZQ Li Y Wang BG Zhang MF. Long Intergenic Noncoding RNA 01296 Aggravates Gastric Cancer Cells Progress Through miR-122/MMP-9. BioMed Pharmacother (2018) 97:450–57. doi: 10.1016/j.biopha.2017.10.066 29091895

[100] Li Y Jiang L Lv S Xu H Fan Z He Y. E2F6-Mediated lncRNA CASC2 Down-Regulation Predicts Poor Prognosis and Promotes Progression in Gastric Carcinoma. Life Sci (2019) 232:116649. doi: 10.1016/j.lfs.2019.116649 31301415

[101] Xu W He L Li Y Tan Y Zhang F Xu H. Silencing of lncRNA ZFAS1 Inhibits Malignancies by Blocking Wnt/beta-Catenin Signaling in Gastric Cancer Cells. Biosci Biotechnol Biochem (2018) 82(3):456–65. doi: 10.1080/09168451.2018.1431518 29424266

[102] Wei R Ding C Rodriguez RA Del Mar Requena Mullor M. The SOX2OT/miR-194-5p Axis Regulates Cell Proliferation and Mobility of Gastric Cancer Through Suppressing Epithelial-Mesenchymal Transition. Oncol Lett (2018) 16(5):6361–68. doi: 10.3892/ol.2018.9433 PMC6202518 30405772

[103] Zhang W Song Y. LINC00473 Predicts Poor Prognosis and Regulates Cell Migration and Invasion in Gastric Cancer. BioMed Pharmacother (2018) 107:1–6. doi: 10.1016/j.biopha.2018.07.061 30071345

[104] Chai F Liang Y Zhang F Wang M Zhong L Jiang J. Systematically Identify Key Genes in Inflammatory and Non-Inflammatory Breast Cancer. Gene (2016) 575(2 Pt 3):600–14. doi: 10.1016/j.gene.2015.09.025 26403314

[105] Sun Q Zhao H Zhang C Hu T Wu J Lin X. Gene Co-Expression Network Reveals Shared Modules Predictive of Stage and Grade in Serous Ovarian Cancers. Oncotarget (2017) 8(26):42983–96. doi: 10.18632/oncotarget.17785 PMC5522121 28562334

[106] Liu W Wei H Gao Z Chen G Liu Y Gao X. COL5A1 may Contribute the Metastasis of Lung Adenocarcinoma. Gene (2018) 665:57–66. doi: 10.1016/j.gene.2018.04.066 29702185

[107] Lal G Hashimi S Smith BJ Lynch CF Zhang L Robinson RA. Extracellular Matrix 1 (ECM1) Expression is a Novel Prognostic Marker for Poor Long-Term Survival in Breast Cancer: A Hospital-Based Cohort Study in Iowa. Ann Surg Oncol (2009) 16(8):2280–7. doi: 10.1245/s10434-009-0533-2 19521735

[108] Chen H Jia WD Li JS Wang W Xu GL Ma JL. Extracellular Matrix Protein 1, a Novel Prognostic Factor, is Associated With Metastatic Potential of Hepatocellular Carcinoma. Med Oncol (2011) 28(Suppl 1):S318–25. doi: 10.1007/s12032-010-9763-1 21128013

[109] Shiga K Hara M Nagasaki T Sato T Takahashi H Takeyama H. Cancer-Associated Fibroblasts: Their Characteristics and Their Roles in Tumor Growth. Cancers (Basel) (2015) 7(4):2443–58. doi: 10.3390/cancers7040902 PMC4695902 26690480

[110] Biffi G Tuveson DA. Diversity and Biology of Cancer-Associated Fibroblasts. Physiol Rev (2021) 101(1):147–76. doi: 10.1152/physrev.00048.2019 PMC7864232 32466724

[111] Minami T Aoyagi K Kawahara A Murakami N Isobe T Tanaka Y. Evaluation of the Expression of Bone Marrow-Derived Mesenchymal Stem Cells and Cancer-Associated Fibroblasts in the Stroma of Gastric Cancer Tissue. Ann Gastroenterol Surg (2020) 4(4):464–74. doi: 10.1002/ags3.12347 PMC7382433 32724891

[112] Erin N Grahovac J Brozovic A Efferth T. Tumor Microenvironment and Epithelial Mesenchymal Transition as Targets to Overcome Tumor Multidrug Resistance. Drug Resist Update (2020) 53:100715. doi: 10.1016/j.drup.2020.100715 32679188

[113] Bu L Baba H Yoshida N Miyake K Yasuda T Uchihara T. Biological Heterogeneity and Versatility of Cancer-Associated Fibroblasts in the Tumor Microenvironment. Oncogene (2019) 38(25):4887–901. doi: 10.1038/s41388-019-0765-y 30816343

[114] Zeng D Li M Zhou R Zhang J Sun H Shi M. Tumor Microenvironment Characterization in Gastric Cancer Identifies Prognostic and Immunotherapeutically Relevant Gene Signatures. Cancer Immunol Res (2019) 7(5):737–50. doi: 10.1158/2326-6066.CIR-18-0436 30842092

[115] Yan Y Wang LF Wang RF. Role of Cancer-Associated Fibroblasts in Invasion and Metastasis of Gastric Cancer. World J Gastroenterol (2015) 21(33):9717–26. doi: 10.3748/wjg.v21.i33.9717 PMC4562955 26361418

[116] Xu G Zhang B Ye J Cao S Shi J Zhao Y. Exosomal miRNA-139 in Cancer-Associated Fibroblasts Inhibits Gastric Cancer Progression by Repressing MMP11 Expression. Int J Biol Sci (2019) 15(11):2320–29. doi: 10.7150/ijbs.33750 PMC6775321 31595150

[117] Barbazan J Matic Vignjevic D. Cancer Associated Fibroblasts: Is the Force the Path to the Dark Side? Curr Opin Cell Biol (2019) 56:71–9. doi: 10.1016/j.ceb.2018.09.002 30308331

[118] Galluzzi L Baehrecke EH Ballabio A Boya P Bravo-San Pedro JM Cecconi F. Molecular Definitions of Autophagy and Related Processes. EMBO J (2017) 36(13):1811–36. doi: 10.15252/embj.201796697 PMC5494474 28596378

[119] Wu X Zhou Z Xu S Liao C Chen X Li B. Extracellular Vesicle Packaged LMP1-Activated Fibroblasts Promote Tumor Progression *via* Autophagy and Stroma-Tumor Metabolism Coupling. Cancer Lett (2020) 478:93–106. doi: 10.1016/j.canlet.2020.03.004 32160975

[120] Wang Z Wang X Zhang T Su L Liu B Zhu Z. LncRNA MALAT1 Promotes Gastric Cancer Progression *via* Inhibiting Autophagic Flux and Inducing Fibroblast Activation. Cell Death Dis (2021) 12(4):368. doi: 10.1038/s41419-020-03316-w 33824303 PMC8024309

[121] Hashimoto T Shibasaki F. Hypoxia-Inducible Factor as an Angiogenic Master Switch. Front Pediatr (2015) 3:33. doi: 10.3389/fped.2015.00033 25964891 PMC4408850

[122] Bahramian S Sahebi R Roohinejad Z Delshad E Javid N Amini A. Low Expression of LncRNA-CAF Attributed to the High Expression of HIF1A in Esophageal Squamous Cell Carcinoma and Gastric Cancer Patients. Mol Biol Rep (2022) 49(2):895–905. doi: 10.1007/s11033-021-06882-0 35040008

[123] Huang C Liu J He L Wang F Xiong B Li Y. The Long Noncoding RNA Noncoding RNA Activated by DNA Damage (NORAD)-microRNA-496-Interleukin-33 Axis Affects Carcinoma-Associated Fibroblasts-Mediated Gastric Cancer Development. Bioengineered (2021) 12(2):11738–55. doi: 10.1080/21655979.2021.2009412 PMC8810175 34895039

[124] Conlon KC Miljkovic MD Waldmann TA. Cytokines in the Treatment of Cancer. J Interferon Cytokine Res (2019) 39(1):6–21. doi: 10.1089/jir.2018.0019 29889594 PMC6350412

[125] Saraiva M Vieira P O'Garra A. Biology and Therapeutic Potential of Interleukin-10. J Exp Med (2020) 217(1):e20190418. doi: 10.1084/jem.20190418 31611251 PMC7037253

[126] Yuan MJ Wang T. Advances of the Interleukin-21 Signaling Pathway in Immunity and Angiogenesis. BioMed Rep (2016) 5(1):3–6. doi: 10.3892/br.2016.665 27330746 PMC4907214

[127] Zhang M Mathews Griner LA Ju W Duveau DY Guha R Petrus MN. Selective Targeting of JAK/STAT Signaling is Potentiated by Bcl-xL Blockade in IL-2-Dependent Adult T-Cell Leukemia. Proc Natl Acad Sci U.S.A. (2015) 112(40):12480–5. doi: 10.1073/pnas.1516208112 PMC4603455 26396258

[128] Yan L Zhang J Guo D Ma J Shui SF Han XW. IL-21R Functions as an Oncogenic Factor and is Regulated by the lncRNA MALAT1/miR-125a-3p Axis in Gastric Cancer. Int J Oncol (2019) 54(1):7–16. doi: 10.3892/ijo.2018.4612 30387833 PMC6255062

[129] Zhou R Wu Z Deng X Chen H. The Long Non-Coding RNA OLC8 Enhances Gastric Cancer by Interaction With IL-11. J Clin Lab Anal (2019) 33(8):e22962. doi: 10.1002/jcla.22962 31273847 PMC6805327

[130] Seoane J Gomis RR. TGF-Beta Family Signaling in Tumor Suppression and Cancer Progression. Cold Spring Harb Perspect Biol (2017) 9(12):a022277. doi: 10.1101/cshperspect.a022277 28246180 PMC5710110

[131] Zhang X Wu J. LINC00665 Promotes Cell Proliferation, Invasion, and Metastasis by Activating the TGF-Beta Pathway in Gastric Cancer. Pathol Res Pract Aug (2021) 224:153492. doi: 10.1016/j.prp.2021.153492 34091388

[132] Zhang Q Chen B Liu P Yang J. XIST Promotes Gastric Cancer (GC) Progression Through TGF-Beta1 *via* Targeting miR-185. J Cell Biochem (2018) 119(3):2787–96. doi: 10.1002/jcb.26447 29053187

[133] Saito T Kurashige J Nambara S Komatsu H Hirata H Ueda M. A Long Non-Coding RNA Activated by Transforming Growth Factor-Beta is an Independent Prognostic Marker of Gastric Cancer. Ann Surg Oncol (2015) 22 Suppl 3:S915–22. doi: 10.1245/s10434-015-4554-8 25986864

[134] Pawluczuk E Lukaszewicz-Zajac M Mroczko B. The Role of Chemokines in the Development of Gastric Cancer - Diagnostic and Therapeutic Implications. Int J Mol Sci (2020) 21(22):8456. doi: 10.3390/ijms21228456 PMC7697532 33182840

[135] Dong XZ Zhao ZR Hu Y Lu YP Liu P Zhang L. LncRNA COL1A1-014 is Involved in the Progression of Gastric Cancer *via* Regulating CXCL12-CXCR4 Axis. Gastric Cancer (2020) 23(2):260–72. doi: 10.1007/s10120-019-01011-0 31650323

